# Monitoring serotonin signaling on a subsecond time scale

**DOI:** 10.3389/fnint.2013.00044

**Published:** 2013-06-05

**Authors:** Elyse C. Dankoski, R. Mark Wightman

**Affiliations:** ^1^Curriculum in Neurobiology, University of North CarolinaChapel Hill, NC, USA; ^2^Department of Chemistry, University of North CarolinaChapel Hill, NC, USA

**Keywords:** 5-HT, cyclic voltammetry, carbon-fiber microelectrode, selective serotonin reuptake inhibitor, serotonin autoreceptor, serotonin transporter

## Abstract

Serotonin modulates a variety of processes throughout the brain, but it is perhaps best known for its involvement in the etiology and treatment of depressive disorders. Microdialysis studies have provided a clear picture of how ambient serotonin levels fluctuate with regard to behavioral states and pharmacological manipulation, and anatomical and electrophysiological studies describe the location and activity of serotonin and its targets. However, few techniques combine the temporal resolution, spatial precision, and chemical selectivity to directly evaluate serotonin release and uptake. Fast-scan cyclic voltammetry (FSCV) is an electrochemical method that can detect minute changes in neurotransmitter concentration on the same temporal and spatial dimensions as extrasynaptic neurotransmission. Subsecond measurements both *in vivo* and in brain slice preparations enable us to tease apart the processes of release and uptake. These studies have particularly highlighted the significance of regulatory mechanisms to proper functioning of the serotonin system. This article will review the findings of FSCV investigations of serotonergic neurotransmission and discuss this technique's potential in future studies of the serotonin system.

## Introduction

The neurotransmitter serotonin [also called 5-hydroxytryptamine (5-HT)] can be found in nearly every region of the central nervous system. Its functions are as diverse as the areas they innervate, and it is a complex component of many psychiatric disorders. This pervasive involvement in brain-wide neurocircuitry is supported by an exceptionally large family of receptors whose collective functional scope enables the multifarious actions of serotonin throughout the brain (Barnes and Sharp, 1999). Much of our knowledge about serotonin comes from studies investigating its actions via these receptors, which remain the target of many pharmacotherapies involving serotonergic signaling. However, the release and uptake dynamics of serotonin precipitate its downstream effects, and exploring how these dynamics are modulated has provided key insights to the serotonin system and its therapeutic potential.

A number of techniques have been used to characterize serotonin signaling in the brain. *In vivo* microdialysis has provided key insights into natural and pharmacologically-induced fluctuations in ambient extracellular levels of serotonin. However, even recent advancements in microdialysis sampling rates provide markedly lower temporal resolution than required to examine individual release and uptake events (Schultz and Kennedy, 2008). Electrophysiological measurements can infer some properties of neurotransmitter release by measuring postsynaptic response, and this method works well for neurotransmitters like glutamate and GABA, whose ligands effect instantaneous changes in ionic current or membrane potential. However, most serotonin receptors in the brain are G-protein coupled and activate intracellular cascades over time periods of 400 ms or more, resulting in postsynaptic effects that are too slow or heterogeneous to reveal information about small, fast changes in concentration. Rigorous characterization of serotonin signaling requires a technique that operates on the same temporal and spatial scales as its release and uptake processes.

Electroanalytical techniques, which combine chemical selectivity with high temporal resolution, are often used in brain tissue to monitor small, fast changes in neurotransmitter concentrations concurrent with release and uptake. Serotonin signaling has been studied using several electroanalytical techniques, including differential pulse voltammetry and chronoamperometry [for a review, see Stamford (1985)]. Among these techniques, fast-scan cyclic voltammetry (FSCV) is the best combination of temporal and chemical sensitivity for measuring endogenous changes in serotonin concentration in brain tissue. This article will review the findings of voltammetric studies and discuss their contribution to current understanding of the mechanisms modulating serotonin release and uptake.

## Fast-scan cyclic voltammetry of serotonin

**Figure 1:**
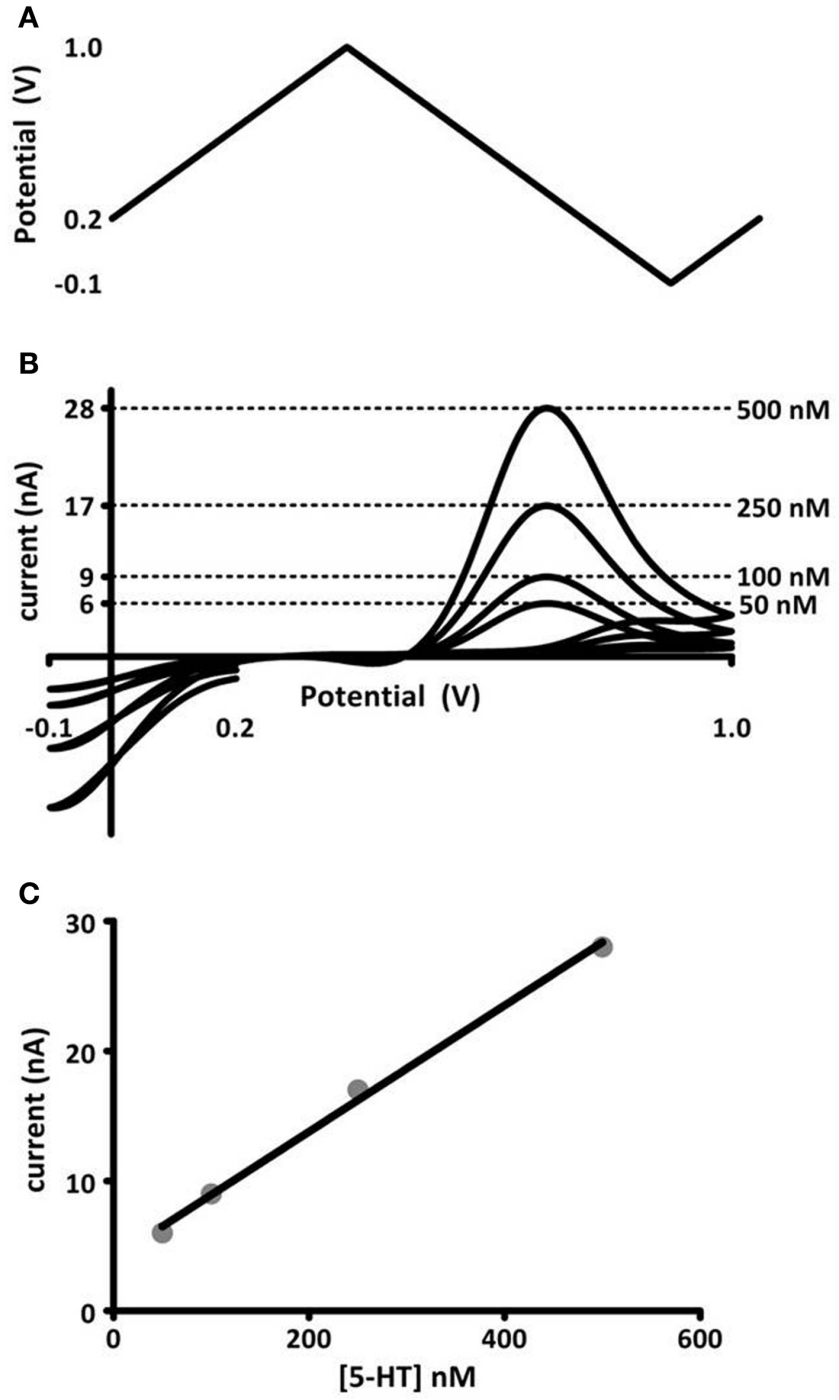
***In vitro* calibration of microelectrodes. (A)** Voltage potential waveform, described by Jackson et al. (1995), for detection of serotonin. **(B)** Cyclic voltammograms (current-voltage curves) obtained for known concentrations of serotonin injected into a flow cell apparatus. The concentration (right) and its corresponding oxidation current amplitude (left axis) are noted by dashed lines. **(C)** Maximal oxidation current vs. concentration of serotonin. The data are fit to a linear regression (black line), the slope of which gives a calibration factor for serotonin measured at these electrodes.

FSCV is an electrochemical technique that detects changes in endogenous neurotransmitter levels rapidly enough to distinguish release and uptake events in brain tissue. The monoamine neurotransmitters dopamine, norepinephrine, and serotonin are well-suited to voltammetric detection because they oxidize predictably and at low potentials. To evaluate changes in neurotransmitter concentration, FSCV measures the current generated by the oxidation of a neurotransmitter. Oxidation is driven by a potential waveform applied to a carbon-fiber sensor. The current generated is proportional to the concentration of analyte at the carbon surface, so the current-to-concentration relationship can be quantified by calibrating microelectrodes in authentic standards before or after experimental use. Chemical selectivity, or the ability to identify the neurotransmitter being measured, is facilitated by analyzing the plot of generated current vs. applied potential. This current-voltage curve is termed the cyclic voltammogram. Monoamines oxidize and reduce at predictable potentials, and their cyclic voltammograms have a characteristic shape that is easy to recognize. An example of a voltage waveform, cyclic voltammograms, and *in vitro* calibration is shown in Figure 1. The “fast-scan” in the technique's name refers to the potential waveform, which is applied rapidly and repeatedly, producing up to 10 cyclic voltammograms per second. The carbon-fiber microelectrode sensors used in FSCV have small dimensions (5 × 100 μm), and this small size enables sampling from as few as 100 synapses at a time, with the electrode targeted to a discrete brain region. Thus, FSCV is a technique for which temporal and spatial scales of data collection are compatible with monitoring neurotransmission.

### Brain regions with measurable serotonin release

In brain slices, changes in serotonin concentration can be evoked using local electrical stimulation in brain regions containing serotonergic neurons or their axonal projections. The dorsal raphe nucleus (DRN), a tiny hub in the core of the medulla, contains the majority of serotonin-producing neurons that send ascending projections into the brain. Voltammetric measurements detect serotonin efflux from both axonal and somatodendritic sites in this region because a subset of serotonergic neurons synapse locally. Although axonal serotonin release is prevalent throughout the central nervous system, experiments employing FSCV are typically constrained to brain regions dense with serotonergic terminals and limited interference from other neurotransmitters and metabolites. These studies predominantly take place in the substantia nigra, a midbrain region composed of the *pars compacta*, packed with dopamine-synthesizing neurons, and the *pars reticulata* (SNr), a networked relay region that includes the densest serotonergic projections from the DRN to any forebrain region (9 × 10^6^ sites per mm^3^) (Moukhles et al., 1997). In the SNr, serotonin is the predominant electroactive neurotransmitter evoked by electrical stimulations, frequently observed in the absence of somatodendritic dopamine release (Cragg et al., 1997). However, Moukhles et al. (1997) reported that serotonergic processes form synaptic junctions at a high rate in the SNr than any other brain region. It should be considered, therefore, that serotonin dynamics described in this region may be dissimilar to the dynamics in other brain regions, including the cerebral cortex, neostriatum, and hippocampus, where a majority of serotonin terminals form non-junctional synapses (Descarries et al., 1990). Serotonin efflux has also been described using FSCV in brain slices containing the suprachiasmatic nucleus (SCN) and ventral lateral geniculate nucleus (vLGN), hypothalamic and thalamic areas with similarly robust serotonergic innervation.

Serotonin measurements in *in vivo* FSCV experiments have taken place exclusively in the SNr. Thick vasculature and meninges above the DRN make targeting this region in the intact brain with a fragile carbon-fiber microelectrode difficult. Other serotonergic regions of interest, such as the hippocampus and prefrontal cortex, have not been explored due to significant chemical interference from other monoamines. However, recent advancements in neuronal stimulation technology may help circumvent this problem, and these potential future directions will be discussed in more detail in the conclusion of this article.

### Electrochemical identification

Electrochemical methods, including FSCV, lack absolute chemical specificity. Some chemical species, particularly those with similar structure, can interfere with detection of the desired substance by oxidizing at similar or identical potentials. Therefore, voltammetric measurements rely on five criteria for identification of endogenously released substances: First, cyclic voltammograms obtained under experimental conditions must have high correlation with cyclic voltammograms of the authentic compound. Second, presence of the neurotransmitter must be validated by independent chemical identification, such as microdialysis, tissue content analysis, or radioligand binding in the targeted brain region. The third criterion requires precise anatomical positioning of the sensor into the brain region of interest. Fourth, observed release should follow known physiological properties for the neurotransmitter and target brain region. Finally, identification of the released substance is dependent on pharmacological validation.

O'Connor and Kruk (1991a) systematically addressed the criteria for electrochemical validation in the first published report of endogenous serotonin measured in rat brain slices using FSCV (O'Connor and Kruk, 1991a). The cyclic voltammogram obtained from electrically-evoked serotonin is highly correlated to the one obtained after adding known concentrations of serotonin to the bath solution. Stimulation trains (500 ms in duration) elicited transient flux in serotonin levels in the DRN, where serotonin-synthesizing neurons are located, and SCN, a region dense in serotonin projections from the DRN (Fuxe, 1965). The evoked concentrations measured in both brain regions was completely and reversibly abolished by removal of calcium from the buffer solution or addition of sodium-channel blocker tetrodotoxin, complying with known physiological properties of exocytotic release. RO 4-1284, an irreversible vesicular monoamine transporter 2 (VMAT2) inhibitor, attenuated release, confirming that observed release was vesicular in nature. Inhibition of monoamine oxidase (MAO) had no effect on stimulated efflux, ruling out interference from serotonin's metabolite, 5-HIAA. Finally, the clearance rate of serotonin in the DRN and SCN could be decreased after application of citalopram, a selective serotonin uptake inhibitor, but not by benztropine, a norepinephrine uptake inhibitor, to bath solution. Similar procedures validate the identity of serotonin detected in subsequent experiments by this and other groups.

**Figure 2:**
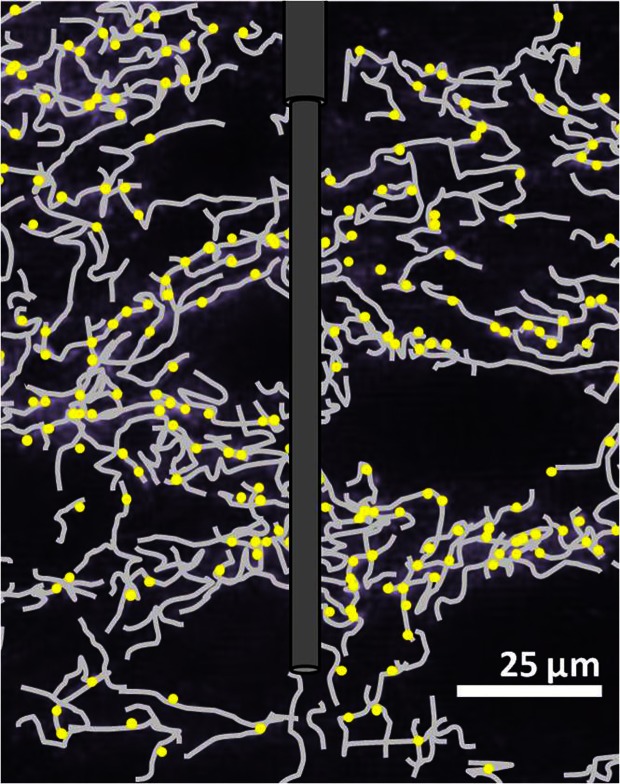
**Illustration of a carbon-fiber microelectrode in SNr**. Scaling of the microelectrode to *in situ* serotonergic fibers (gray) and uptake sites (yellow) is a representation based on Moukhles et al. (1997).

Bunin and Wightman (1998) later investigated an aspect of serotonin's physiological release properties that had not been addressed by initial voltammetric characterizations. The dimensions of carbon-fiber microelectrodes are considerably larger than the synaptic cleft into which neurotransmitters are released (Figure 2). Consequently, FSCV detects extracellular, not intrasynaptic, changes in concentration, and its measurements are limited to the neurotransmitter concentration that diffuses into the extrasynaptic space following release. A number of neuromodulators diffuse beyond the synaptic space to reach their receptors and transporters in a process called volume transmission (Fuxe et al., 2010), and prior evidence from non-voltammetric techniques implicated serotonin as a volume neurotransmitter. Ultrastructural studies of serotonergic terminals throughout the brain suggest that they form predominantly non-junctional synapses (Chazal and Ralston, 1987). This terminal architecture, together with reports that expression of serotonin transporters and receptors occurs primarily on extrasynaptic regions of neuronal processes (Kia et al., 1996; Zhou et al., 1998), is indicative of volume transmission. In light of this information, Bunin and Wightman (1998) hypothesized that electrically-evoked serotonin should reach the extracellular space via diffusion, without buffering from uptake and receptor binding sites. This was found to be the case for both somatodendritic and terminal release, where the concentration of serotonin evoked per stimulation pulse during 20-pulse trains was equivalent to the concentration evoked by a single pulse (Bunin and Wightman, 1998). Therefore, the authors concluded that serotonin concentrations measured by voltammetry reflect physiological volume transmission from the synapse to its extrasynaptic targets.

### Technical considerations

Since O'Connor and Kruk's first report of voltammetric detection of serotonin, several modifications have been implemented to adapt and improve the use of FSCV for novel applications. The voltage potential waveform (−1 to +1.4 to −1) used by the Stamford and Kruk labs, as well as others, in serotonin studies cited throughout this review was adjusted by Jackson et al. (1995) to improve temporal resolution. This modification to an N-shaped waveform, which scans from +0.2 to +1.0 to −0.1 back to +0.2 (Figure 1A), was designed to reduce serotonin adsorption to the electrode surface, as this slows electrode response times. It also avoids fouling reactions of serotonin's oxidative and reductive byproducts, improving electrode sensitivity and stability over time (Jackson et al., 1995). The modified waveform's improvement enhanced electrode response times and enabled more accurate measurements of release and uptake rates, facilitating closer examination of the kinetic parameters of serotonin release.

Improvement of carbon-fiber microelectrode sensors has been another ongoing adaptation to voltammetric measurements of serotonin. Brazell et al. (1987) first reported that dip-coating a carbon-fiber microelectrode in Nafion, a cation-selective polymer, improves serotonin and dopamine detection (Brazell et al., 1987; Jackson et al., 1995). Nafion enhances serotonin detection in two ways: first, by directly increasing the electrode's sensitivity to (positively-charged) serotonin, and second, by reducing its sensitivity to interfering anionic species such as uric acid and serotonin's metabolites. Years later, the success of the first *in vivo* voltammetric measurements of endogenous serotonin concentrations in a rat owe their success to the enhanced sensitivity and temporal resolution facilitated by Nafion-coated sensors and the modified voltage potential waveform (Hashemi et al., 2009).

Many labs continued their investigations of serotonin release without adopting either modification. Because each study reports on electrically-evoked changes in serotonin concentration, which are derived from *in vitro* calibrations, comparing findings between labs is not considered an issue within this review. It is important to note that these calibrations do not take into account the deleterious effects of electrode fouling that may be appreciably different over the course an experiment depending on the waveform used. Regardless of waveform choice, however, demonstration of a linear relationship between concentration applied and the current evoked establish the suitability of a waveform for stable detection of serotonin.

## Electrical stimulation

Many of the optimal electrical stimulation parameters for evoking somatodendritic and terminal serotonin in brain slices are consistent with previously established physiological principles. Serotonergic fibers are not myelinated and, like other unmyelinated fibers, are maximally excited by wider stimulation pulse widths, up to 2 ms in length (Anden et al., 1967; Merrill et al., 1978; Millar et al., 1985; Bunin et al., 1998). The amplitude of evoked serotonin concentration is also strongly dependent on increases in stimulation intensity (up to 380 μA) and number of pulses in a stimulation train. Maximal release amplitudes are also positively correlated with increasing frequency, up to 100 Hz (O'Connor and Kruk, 1991b; Iravani and Kruk, 1997; Bunin and Wightman, 1998), although a detailed investigation by John et al. (2006) found that electrically-evoked concentrations were less sensitive to stimulation frequencies above 30 Hz (John et al., 2006). These constrained ranges of frequency dependence could reflect limitations in vesicular availability, but Aghajanian et al. (1990) has posited that processes in terminal regions store enough serotonin to sustain long, high frequency release (Aghajanian et al., 1990). Although serotonergic neurons are typically thought to fire at a rate of 0.5–5 Hz, burst-firing in the DRN has been measured at a rate of 100 Hz (Aghajanian et al., 1978; Vandermaelen and Aghajanian, 1983; Hajos et al., 1995). Differences in the range of frequency sensitivity between studies may therefore reflect dynamic, physiological fluctuations and could point to yet another regulatory component within the serotonin system. Future investigation of the mechanisms influencing frequency dependence would be an interesting addition to our understanding of serotonin signaling.

Although voltammetric studies of serotonin have used a wide array of stimulation parameters, one type has been used repeatedly in the studies reviewed in this article. Pseudo-one-pulse (POP) stimulations consist of 5–10 pulses applied at 100–200 Hz and are shorter than 100 ms in duration. They are designed to approximate a single electrical impulse but evoke more consistent efflux. In brain slice experiments, POP stimulations are often used to avoid creating an endogenous “tone” at receptors, which facilitates more direct investigation of selective agonists and antagonist's effects on autoreceptor-mediated modulation of release (Limberger et al., 1991; Thienprasert and Singer, 1993).

Endogenous serotonin concentrations have been evoked *in vivo* using electrical stimulation of the DRN as well as the medial forebrain bundle (MFB). A subset of serotonergic neurons that project to the SNr also send axon collaterals to forebrain structures via the MFB (Van Der Kooy and Hattori, 1980). Electrical stimulation of these collaterals excites SNr-projecting neurons in a retrograde direction, eliciting serotonin in the desired region (Hashemi et al., 2011). While targeting a stimulation electrode to the MFB is less challenging than targeting the DRN, this stimulation site can also be used to evoke neurotransmitter release in many brain regions. This may have indirect effects on serotonin signaling, complicating interpretation of data. Many optimal stimulation parameters are consistent between *in vitro* and *in vivo* measurements, including pulse width, stimulation intensity, and stimulation length. However, the concentration of serotonin evoked in the SNr is remarkably lower than predicted by brain slice measurements, prompting curiosity about the potential for serotonergic regulatory mechanisms that require intact brain tissue.

## Release

Local electrical stimulations of serotonin terminals in brain slices typically evoke concentration changes in the 100 nM range. *In vivo*, however, serotonin concentrations evoked in the SNr rarely reach 100 nM, even after pharmacological manipulations (Hashemi et al., 2012). *In vivo* serotonin release, measured in an intact brain, is presumably limited by negative feedback from somatodendritic and terminal autoreceptors as well as inhibitory neurotransmitters that are released concurrently, which may account for some of the disparity in release amplitudes. In brain slices, concentration flux coincides with onset of the stimulation pulse train and this rising phase reaches its maximum within milliseconds of the stimulation's end. Serotonin evoked *in vivo* tends to overshoot the duration of stimulation. The overshoot is partially an effect of the broader area of release sites activated by a remote stimulation location, but is also due to limited diffusion rates through a Nafion polymer coating that is applied to enhance sensitivity *in vivo* (Hashemi et al., 2009).

As mentioned in a previous section, electrically-evoked serotonin concentrations measured in brain slices are sensitive to stimulation frequency. A proposal by Wightman et al. (1988) explains this observation: more uptake occurs in the time between stimulation pulses during low frequency stimulations, which limits the summation of extracellular neurotransmitter concentration (Wightman et al., 1988). Jennings et al. (2010) hypothesized that shifts in uptake rate associated with differential serotonin transporter expression would predictably alter this frequency dependence. Mice with either gain or loss of SERT expression both displayed significantly lower sensitivity to stimulation frequency than their wild-type littermates. Furthermore, in wild-type mice, a selective serotonin transporter inhibitor reduced sensitivity to stimulation frequency (Jennings et al., 2010). These findings underscore the importance of SERT in establishing a functional, dynamic equilibrium between release and uptake that enables coherent serotonin signaling.

**Figure 3:**
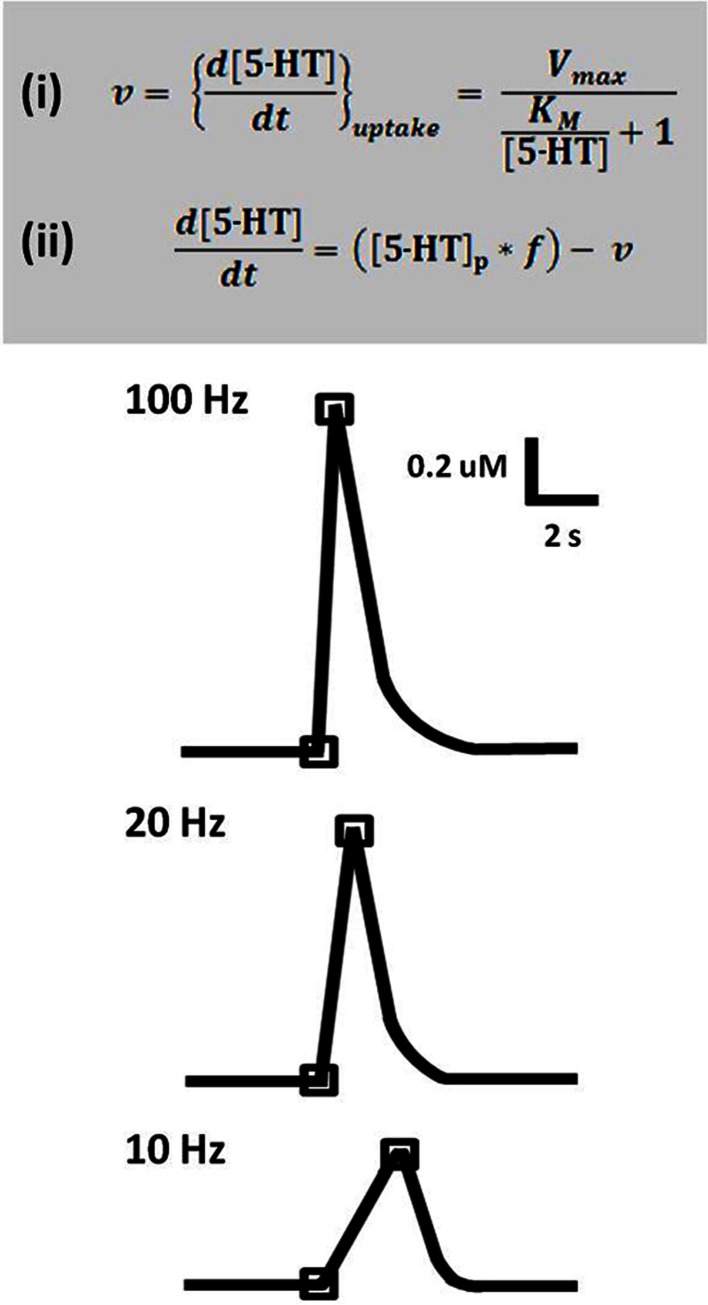
**A model predicts serotonin release and uptake at different frequencies. Top panel:** Equation **(i)** describes rate of uptake (*v*) as a function of maximal uptake rate (*V*_max_), the Michaelis constant for SERT (*K_M_*), and concentration of released serotonin ([5-HT]). Equation **(ii)** describes the expected concentration of released serotonin given a stimulation with number of pulses (p) and frequency (*f*), less the uptake that occurs over this time period. **Lower panel:** Concentration evoked by 50 pulse stimulation trains at 100, 20, and 10 Hz, as predicted by the model. Traces are representations of data based on Bunin et al. (1998).

**Figure 4:**
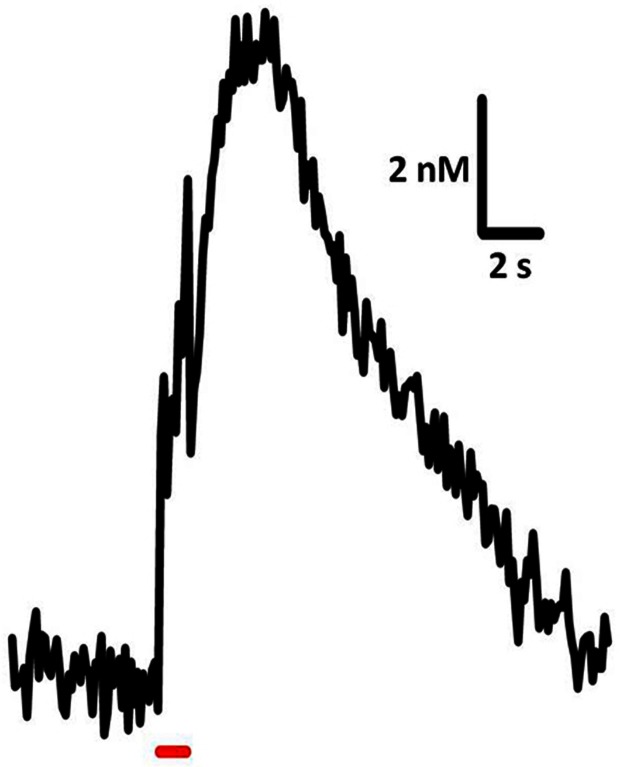
**Sample trace of *in vivo* serotonin release and uptake**. Release was elicited using 60 pulses, 60 Hz, 325 μA stimulation of the DRN and recorded in mouse SNr. Average of signals from 7 subjects.

Time-resolved measurements with FSCV also enable examination and comparison of the kinetic parameters of serotonin transmission. Neurotransmitter uptake is assumed to follow Michaelis–Menten dynamics, and uptake as well as concentration evoked per stimulus pulse can be calculated using a modified model of enzyme kinetics. Figure 3 shows the equations used to model (**i**) uptake and (**ii**) release and representative signals predicted for stimulations of varying frequency. In brain slice preparations, the concentration evoked per stimulation pulse ([5-HT]_pulse_) was found to be 100 ± 20 nM in DRN, and significantly lower in the SNr, at 55 ± 7 nM. Differences in [5-HT]_pulse_ are proportional to differences in tissue content between the two brain regions, indicating that local stores of serotonin may influence the concentration that can be evoked by electrical stimulation (Bunin et al., 1998). *In vivo* [5-HT]_pulse_ in the SNr is much lower, comparatively: 1.5 nM per pulse using DRN stimulation, and 1.1 nM per pulse from the MFB. Figure 4 shows an averaged recording of *in vivo* serotonin signals in the SNr; note that the concentration evoked is strikingly lower than predicted by the model in Figure 3. Given that both Bunin et al. (1998) and Hashemi et al. (2011) conducted experiments in the SNr, the nearly 50-fold difference cannot be attributed to differences in tissue content. Instead, this discrepancy between brain slice and *in vivo* preparations suggests powerful regulatory mechanisms acting on serotonin release *in vivo* which may depend on intact circuitry.

Hashemi et al. (2012) investigated mechanisms that may limit *in vivo* neurotransmission by using a common MFB stimulation to compare serotonin and dopamine efflux in the SNr and nucleus accumbens, respectively. The dopamine system serves as a good basis for comparison with the serotonin system because the two monoamines share parallel features in the mechanisms controlling their synthesis, release, modulation, uptake, and metabolic degradation. Inhibition of the monoamine synthesis enzyme aromatic amino acid decarboxylase and monoamine vesicular packing protein (VMAT2) considerably decreased the concentration of evoked dopamine to 18% and 6% of control amplitudes, respectively, but affected serotonin to a much lesser extent (48% and 72%, respectively). Serotonin efflux was also resistant to short term depression after repeated stimulation pulse trains, while dopamine efflux was attenuated by 38% after 20 stimulations. This suggests that a relatively small proportion of the available vesicular serotonin is mobilized for release by each electrical stimulation train, a finding which may partially explain the low concentrations observed *in vivo*.

### Modulation by autoreceptors

Three subtypes of serotonin receptors, all 5-HT1-type, are expressed on serotonergic axons, soma, and dendrites and function as autoreceptors that provide inhibitory feedback. 5-HT1-type receptors are found throughout the brain as autoreceptors, expressed on pre-synaptic serotonin terminals, and also as heteroreceptors, expressed on post-synaptic targets. The most well-studied autoreceptors, 5-HT1A, 1B, and 1D are seven transmembrane, G-protein coupled receptors (GPCRs). 5-HT1B and 1D autoreceptors negatively couple to adenylyl cyclase (Yocca and Maayani, 1990). 5-HT1A heteroreceptors throughout the brain also inhibit adenylyl cyclase activity, but autoreceptors in the DRN apparently function through a different G_i_-coupled mechanism (Clarke et al., 1996). *In vivo* studies of these autoreceptors are challenging because even highly-selective drugs inadvertently target pharmacologically-identical heterosynaptic receptors, which are often expressed at high levels in the same brain region as the autoreceptor. Intact circuitry thus makes it difficult to extricate direct effects of autoreceptor activity from indirect regulation by heteroreceptors.

Voltammetric measurements in brain slices avoid some of the problems associated with 5-HT1-type receptor pharmacology. In slices, the absence of spontaneous activity in serotonergic cells, due to either separation from cell bodies in a terminal slice or elimination of noradrenergic inputs in a DRN slice, results in loss of endogenous serotonin tone (Judge and Gartside, 2006). Therefore, these experiments avoid tonic activation of autoreceptors and can also avoid transient autoreceptor activity, when appropriate, using POP stimulations. This provides an opportunity to study the timing and function of these receptors in relative isolation. O'Connor and Kruk (1991b) showed that the non-selective autoreceptor antagonist methiothepin did not affect the concentration of serotonin evoked by POP stimulations, but increased serotonin elicited by longer stimulations. Further exploration with stimulations of varying frequency and duration determined that activation of autoreceptors requires a stimulation period of at least 400 ms (O'Connor and Kruk, 1991b). This time frame is comparable to the activation window for dopamine autoreceptors in striatal and limbic regions. Phillips et al. (2002) found that the activation delay observed for dopamine autoreceptors reflects timing of intracellular cascades added to the rate of neurotransmitter diffusion in a given brain area (Phillips et al., 2002).

5-HT1A, 1B, and 1D receptors are expressed at high levels in the DRN, where they negatively influence neuronal firing rate and extracellular levels of serotonin (Sprouse and Aghajanian, 1987; Pineyro et al., 1995; Moret and Briley, 1997; Adell et al., 2001). Voltammetric studies corroborate the inhibitory functions of all three receptors in this region by demonstrating that their selective agonists can reduce the amplitude of electrically-evoked serotonin release (Davidson and Stamford, 1995b; Hopwood and Stamford, 2001). Although its heteroreceptor analogues are prominently expressed in limbic regions, 5-HT1A autoreceptors are only expressed in the DRN and median raphe nucleus (Verge et al., 1985). Serotonin levels in forebrain terminal regions are affected by 5-HT1A-mediated changes in DRN unit activity (Kreiss and Lucki, 1994; Casanovas et al., 1997), but only 5-HT1B and 1D autoreceptors are expressed locally to functionally inhibit release in these regions. Voltammetric measurements in the SCN and vLGN confirm absence of 5-HT1A autoreceptor function in these terminal regions. 5-HT1B and 1D receptors, and not 5-HT1A receptors, negatively influence serotonin efflux in brain slices containing terminal regions (O'Connor and Kruk, 1992; Davidson and Stamford, 1996).

The 5-HT1A receptor may be the trump card in this family of autoreceptors: 5-HT1A receptor mRNA is expressed in nearly 100% of serotonergic cells and up to 15% of GABAergic interneurons in the DRN (Day et al., 2004). This receptor robustly regulates both neuronal firing rates and extracellular serotonin levels in the DRN (Sprouse and Aghajanian, 1987; Hjorth and Sharp, 1991). Voltammetric measurements find that antagonists for 5-HT1A and 1B have a supra-additive effect when administered together: increase in serotonin efflux is greater when both receptors are blocked than would be expected given the effect of each antagonist alone (Roberts and Price, 2001). In addition, the effects of 5-HT1B receptor antagonists on serotonin efflux are overpowered by 5-HT1A receptors unless they are also blocked, suggesting that these receptors compensate for reductions of 5-HT1B activity. Given these results, it is suggested that 5-HT1A and 1B receptors exhibit a functional interaction that is facilitated by proximal expression sites on serotonin neurons. Interest in 5-HT1A receptors has increased in the last decade, as they may play a role in depression and anxiety-related disorders (Ohno, 2010). Use of FSCV in future studies could meaningfully contribute to our understanding of how 5-HT1A receptor-mediated modulation of serotonin release plays a role in the etiology and treatment of these disorders.

Much speculation has occurred regarding the explanation for seemingly parallel functions of 5-HT1B and 1D autoreceptors. Both receptors are expressed in most serotonergic brain regions and have superficially redundant effects. One theory posits that these autoreceptors differ in their affinity for serotonin: one high affinity and the other low affinity. However, it has since been demonstrated that their affinities are nearly identical (Boess and Martin, 1994). More likely, the two receptors are expressed in different anatomical locations, and thus provide site-specific regulation of serotonin release, e.g., dendritic vs. axonal localizations in the DRN. Stamford et al. (2000) have reviewed the evidence supporting this hypothesis (Stamford et al., 2000).

The SNr expresses the highest concentration of 5-HT1B autoreceptors and heteroreceptors in the murine brain (Pazos and Palacios, 1985). 5,7-HT-induced lesions of serotonin neurons reduced 5-HT1B expression level by 37%, presumably due to degradation of serotonin terminals (Verge et al., 1986); this suggests that over 1/3 of 5-HT1B receptors expressed in the SNr could function as autoreceptors. Heterosynaptic function of 5-HT1B receptors on presynaptic sites in the SNr has been well-documented and may yield important therapeutic findings (Sari, 2004), but its functionality as an autoreceptor in the SNr remains controversial. Iravani and Kruk (1997) found no effects of 5-HT1B receptor antagonists on electrically-evoked serotonin concentrations in SNr slice preparations (Iravani and Kruk, 1997). However, Threlfell et al. (2010) report that these autoreceptors influence short-term depression of serotonin efflux. In paired stimulation trains, the concentration of serotonin evoked by the second stimulation (S2) reached 30% of that evoked by the first stimulation (S1) when there was a 1 second delay between S1 and S2. Antagonists of 5-HT1B receptors relieved this depression by up to 20% (Threlfell et al., 2010). 5-HT1B autoreceptors are thus apparently functional in the SNr, although their modulatory effects may be less robust than in other brain regions.

It is possible that the role of autoreceptors could be better elucidated by *in vivo* voltammetric studies, where endogenous serotonin tone is undisturbed and autoreceptor function is closer to normal physiological levels. However, a limited number of studies currently address the effects of serotonin's autoreceptors *in vivo*. In practice, it is difficult to selectively target 5-HT1-type receptors on serotonin terminals when pharmacologically indistinct 5-HT1-type heteroreceptors are expressed throughout the brain. As with *in vivo* microdialysis, the direct roles of the autoreceptor would be difficult to extricate from indirect modulation by *in situ* circuitry. Recent technological advancements in iontophoresis enable spatially-resolved, quantitative drug delivery at the site of voltammetric measurements (Herr and Wightman, 2013). Future studies using FSCV combined with this drug-delivery method have great potential to answer important questions about serotonin's autoreceptors.

## Uptake

Serotonin clearance is achieved primarily via active transport. Its transporter, SERT, is a member of the Na^+^/Cl^−^ transporter family, which includes dopamine, norepinephrine, GABA, and glutamate transporters (Bennett et al., 1973; Iversen, 1974). SERT displays high affinity for serotonin in the nanomolar concentration range (Blakely et al., 1991). Inhibitors of SERT, selective serotonin reuptake inhibitors (SSRIs), have been a significant target of research efforts for decades, owing to their widespread use as antidepressant medications. Given acutely, SSRIs exert striking effects on the serotonin system: they elevate extracellular serotonin levels in the DRN (Bel and Artigas, 1992), which in turn decreases rate of cell firing due to activation of 5-HT1A autoreceptors (Chaput et al., 1986; Gartside et al., 1995). However, in therapeutic practice, SSRIs relieve depressive symptoms only after a chronic period of 3–6 weeks. It is during this period that the effects of transport inhibition on serotonin transmission become less clear. FSCV provides an ideal method for deciphering the effects of SSRIs because it can distinguish between changes in released serotonin and changes in rate of uptake.

Electrically-evoked changes in serotonin concentration are cleared from the extrasynaptic space within seconds of stimulation termination. The term *t*_1/2_ is often used to compare rates of clearance; *t*_1/2_ is the time elapsed between peak concentration of neurotransmitter and its decay to half this amplitude. Across brain regions, brain slice and *in vivo* voltammetric measurements report similar values of *t*_1/2_ ranging from approximately 1 to 3 seconds (O'Connor and Kruk, 1994; Iravani et al., 1999; Davidson and Stamford, 2000; Hashemi et al., 2012). Rates of neurotransmitter clearance may positively correlate with the density of transporter sites in a given brain region: Bunin et al. (1998) report clearance rates of 1300 ± 20 nM/s in the DRN and 570 ± 70 nM/s in the SNr, and quantitative autoradiographic studies report 2–4-fold greater SERT binding levels in the DRN (Kovachich et al., 1988; Kovacevic et al., 2010). However, some comparisons of SERT density across brain regions do not support this conclusion, particularly in species other than rat, so more thorough investigation of the relationship between transporter expression and SERT density is needed. In addition to influencing uptake rate, brain slice studies in mice that either lack or overexpress SERT have demonstrated a negative correlation between transporter expression level and concentration of serotonin evoked by electrical stimulation (John et al., 2006; Jennings et al., 2010). The disparity observed between clearance rates in the DRN and SNR is conspicuously proportional to differences Bunin et al. (1998) reported in release rates. This suggests a consistent relationship between transporter expression levels, uptake rates, and release rate. Modeling serotonin signaling kinetics in more brain regions could confirm whether this relationship holds true throughout the brain.

SSRIs decrease rate of neurotransmitter clearance while increasing the maximum amplitude of electrically-evoked serotonin concentrations. In brain slices, SERT inhibition slows clearance (measured as an increase in *t*_1/2_) by 150–700%. This wide spread of responses may be attributable to experimental variability between studies, particularly differences in stimulation parameters. Indeed, *in vivo* studies of SSRI effects in the SNr using identical stimulation parameters report comparable changes in *t*_1/2_ using MFB and DRN stimulation sites (increasing by 324 and 306%, respectively) (Hashemi et al., 2009, 2012). SSRIs also increase evoked serotonin concentrations by 200–450% in SNr brain slices, and up to 410% *in vivo* (Iravani et al., 1999; John et al., 2006; Hashemi et al., 2012). In this case, the intensity of the SSRI's effect is associated with different stimulation frequencies or pulse number. Structure and selectivity differences between SERT inhibitors may also contribute to variable responses between voltammetric studies; however, differences between SSRIs have not been specifically investigated using FSCV. Serotonin efflux in SNr brain slices has been modeled to describe the effects of an SSRI, fluoxetine, on apparent *K_M_*, the Michaelis–Menten constant (John et al., 2006). Quantifying changes to *K_M_, V*_max_ and [5-HT]_p_ may be a more effective way to contrast the effects of various SERT inhibitors on serotonin signaling in future studies in brain slices and *in vivo*. Thorough comparison of these effects could inform clinical usage of these pharmacotherapies.

### Autoreceptors mediate some effects of acute uptake inhibition

In addition to their inhibitory influence on release, serotonin's autoreceptors appear to modulate response to SERT inhibition. A number of studies report that autoreceptor antagonists can potentiate the rise in extracellular serotonin levels elicited by SSRIs (Hjorth, 1993; Artigas et al., 1994), and 5-HT1A autoreceptors also mediate reduction of firing rate by SSRIs in the DRN (Gartside et al., 1995). The concentration change evoked by POP stimulations, deliberately rapid enough to avoid creating an endogenous tone, typically does not activate autoreceptors and is thus not affected by their antagonists. However, in brain slices of the DRN, paroxetine-induced increases in serotonin efflux were potentiated by 5-HT1A and 1B/D receptor antagonists (Davidson and Stamford, 1995a). Therefore, it is hypothesized that SERT inhibition causes an increase in extracellular serotonin levels sufficient to activate autoreceptors, even in brain slices. This produces an inhibitory tone, such that autoreceptor antagonists can further unmask SSRI-induced increases in release. 5-HT1B and 1D autoreceptors appear to similarly potentiate the effects of SSRIs in distal brain regions, as the Stamford lab also reports increases in paroxetine's effects in the vLGN when co-administered with 5-HT1B and 1D receptor antagonists (Davidson and Stamford, 1997). The interaction between regulation of release and uptake functions may also be an important detail in understanding how chronic uptake inhibition functions in treating depressive disorders.

### Chronic uptake inhibition

The gap between onset of acute physiological effects and the therapeutic efficacy achieved in a chronic treatment period implies that SSRI-induced increases in serotonin levels are not directly producing antidepressant effects. Instead, elevated serotonin levels may influence long-term changes in serotonin signaling and its downstream targets to relieve symptoms of depression (Blier et al., 1987). The effects of long-term SERT inhibition are conflicting: some find increases in extracellular serotonin levels, and some find no changes. Associated with these outcomes are variable reports of autoreceptor desensitization or hypersensitization of 5-HT1A and 1B autoreceptors (Chaput et al., 1986; Invernizzi et al., 1992, 1995; Bosker et al., 1995a,b; Moret and Briley, 1996). Studies examining the effects of chronic SSRI treatment using FSCV have produced more consistent findings.

FSCV measurements of serotonin signaling after 21 days of SSRI exposure reveal that rate of clearance, measured by *t*_1/2_, is unchanged by this treatment. This lack of change is intriguing because radioligand binding studies report brain-wide reductions in SERT density after chronic inhibition (Kovacevic et al., 2010). It may reflect compensation by other clearance mechanisms, such as low affinity serotonin transporters. High and low affinity transport systems have been described for other monoamine neurotransmitters (Iversen, 1974; Stamford et al., 1984, 1990; Hagan et al., 2011). Although studies suggest that these transporters may play an important role in serotonin signaling (Daws, 2009), there are presently no FSCV studies describing their role in modulating release and uptake. The role of non-selective uptake transporters in modulating serotonin signaling, particularly following chronic SSRI treatment, would be interesting to investigate using FSCV.

Long-term SSRI treatment increases stimulation-evoked serotonin concentrations in the DRN and other brain regions by 20–100%, depending on the experiment and brain region studied (O'Connor and Kruk, 1994; Davidson and Stamford, 1998, 2000). These findings concur with the results of Schoups et al. (1986), who found that electrically-evoked release of tritiated serotonin (^3^[H]5-HT) in the hypothalamus increased after 21 days of SSRI treatment (Schoups et al., 1986). Although increases in serotonin efflux are observed after acute SERT inhibition, these can be explained by changes in rate of uptake. However, *t*_1/2_ was not altered in any voltammetric investigation of long-term SSRI treatment. Therefore, increases in evoked concentrations induced by chronic treatment must rely on another mechanism. Changes in other aspects of release may contribute to this effect, for example: the quantity or composition of serotonin stored in vesicles, regulation of intracellular calcium, or excitability of the synaptic membrane. In-depth exploration of these mechanisms has not yet been explored using voltammetric methods.

Alterations in 5-HT1A autoreceptors contribute to the effects of chronic SSRIs on serotonin signaling. Under normal conditions, activated 5-HT1A receptors inhibit serotonin release and neuronal firing rates, and chronic SSRI treatment may modify this activity. Selective suppression of 5-HT1A autoreceptors can produce antidepressant behavioral effects in the absence of SSRIs (Bortolozzi et al., 2012). Many investigations have described functional desensitization of 5-HT1A receptors after chronic SERT inhibition, but to varying degrees across brain regions (Kreiss and Lucki, 1994, 1997; Cremers et al., 2000; Bosker et al., 2001; Rossi et al., 2008). Davidson and Stamford (1998) compared serotonin release and uptake and neuronal firing rates in the DRN of rats treated with water or paroxetine for 21 days. Paroxetine-treated rats had significantly higher serotonin release rates but exhibited no differences in firing rate. Interestingly, application of a 5-HT1A receptor agonist revealed that firing rate was *less* sensitive, and release amplitude *more* sensitive, to this manipulation. Contradictory findings of 5-HT1A receptor sensitivity was not a total surprise: prior studies found similar desensitization of 5-HT1A receptors in the control of firing rate after chronic paroxetine treatment (Chaput et al., 1986; Blier et al., 1988, 1990), and O'Connor and Kruk (1994) had previously reported sensitization of 5-HT1A receptors controlling release amplitude. The dichotomous effect of chronic SSRIs on 5-HT1A receptor sensitization indicates a functional distinction between the receptors mediating neuronal firing and those controlling release. Given the complex effects of chronic SSRIs on 5-HT1A autoreceptors in the DRN, it would be interesting to see how these changes translate to serotonin release in an intact brain. Currently, however, no studies employing FSCV have examined the effects chronic SERT inhibition *in vivo*.

5-HT1B and 1D receptors also desensitize after chronic SERT inhibition, although the extent to which this occurs appears to vary between brain regions. O'Connor and Kruk (1994) reported desensitization of 5-HT1B receptors in SCN after chronic treatment with fluoxetine. In contrast, the Stamford lab found no changes in the sensitivity of 5-HT1B receptors in the vLGN, instead finding desensitization of 5-HT1D receptors after chronic paroxetine. This inconsistency may reflect differences in autoreceptor expression in the SCN and vLGN, or result from difficulty in selectively targeting the 5-HT1B receptor pharmacologically (O'Connor and Kruk do not address the effects of 5-HT1D receptors in their study). Additionally, while O'Connor and Kruk (1994) found no desensitization of 5-HT1B receptors in the DRN, Davidson and Stamford (2000) later demonstrated that 5-HT1B receptor desensitization was apparent only when the 5-HT1A autoreceptor is antagonized (Davidson and Stamford, 2000). This adds further weight to the conjecture that 5-HT1A and 1B receptors functionally interact in the DRN.

### Monoamine oxidase

Metabolic degradation of serotonin by the enzyme MAO also contributes to serotonin clearance, especially in the developing brain (Cases et al., 1995, 1998). However, MAO inhibition in brain slices has no reported effect on release amplitudes or uptake (O'Connor and Kruk, 1991a), a finding used to confirm absence of serotonin's metabolites from the voltammetric signal. Owesson et al. (2002) showed a greater role of MAO in regulating serotonin efflux using transgenic mice lacking MAO-A expression. MAO-A is the isoenzyme that preferentially degrades norepinephrine, epinephrine, dopamine, and serotonin, and mice lacking this enzyme have decreased neuronal firing rates in the DRN and increased extracellular serotonin levels (Evrard et al., 2002). In brain slices of the DRN, MAO-A-deficient mice displayed significantly greater serotonin efflux and reduced clearance rates compared to wild-type controls. Additionally, the effects of citalopram were smaller and radioligand binding showed significantly lower expression of SERT in these mice (Owesson et al., 2002). This suggests that serotonin signaling is subject to regulation by MAO under the right experimental conditions. *In vivo* work supports this idea, as a recent study has shown that MAO inhibitors dramatically increase serotonin efflux in the SNr (Hashemi et al., 2012). MAO inhibition also has a much greater effect on serotonin than dopamine efflux when compared *in vivo*, suggesting a unique role for metabolic degradation in the regulation of serotonin transmission compared to other monoaminergic systems.

## Future directions

Most of the studies reviewed in this article focused on describing the role of autoreceptors and transporters in modulating serotonin signaling throughout the brain. However, signaling is also considerably influenced by many other neurotransmitter systems, including norepinephrine, glutamate, GABA, and a number of neuroendocrine modulators. These external influences are highly implicated in serotonin's involvement in a number of psychiatric disorders and, while they have been investigated by other techniques, their functions have not been fully described using subsecond voltammetric measurements. Evaluating the effects of external modulatory mechanisms on the subsecond dynamics of serotonin signaling could provide important clues about their role in neurological disorders.

Ongoing methodological developments continue to progress voltammetric measurements beyond the current experimental limits. While electrochemical techniques have been optimized for serotonin detection (Lama et al., 2012), multi-electrode arrays are being developed which would enable measurements of multiple neurotransmitters in multiple locations simultaneously. Additionally, iontophoretic methods adapted for FSCV now enable localized, quantitative drug delivery, enabling investigation of recording-site specific effects *in vivo*. FSCV can also be paired with concurrent electrophysiological measurements to couple information about neurotransmitter release to single-unit responses of post-synaptic neurons. Iontophoretic and electrophysiological methods have already been applied to voltammetric studies of dopamine release in anaesthetized and freely-moving animals (Takmakov et al., 2011), and the Wightman group is currently working to adapt these methods for serotonin detection.

It has previously been challenging to selectively study serotonin's autoreceptors *in vivo* because homologous receptors are expressed throughout the brain. However, many novel drug-delivery and transgenic methods have been developed to avoid this type of complication. DREADDs, designer receptors with exogenous ligands, have been used to target specific G-protein-activated cascades in serotonergic neurons (Dong et al., 2010). A light-activated 5-HT1A receptor has been generated that can be expressed selectively on serotonergic neurons (Oh et al., 2010). Furthermore, transgenic mice and rats offer many opportunities to study signaling in models of neurological disorders and targeted deletions. The effects of SERT deletion or overexpression on serotonin signaling have been investigated in brain slices of the SNr but not in an *in vivo* preparation. Many conditional knockout mouse models, which avoid confounding developmental effects, are now available for serotonin's transporter and receptors. These techniques could lead to more selective targeting and better characterization of serotonin's receptors and their downstream effectors in combination with voltammetric measurements.

Voltammetric measurements have, until recently, been limited to brain regions with high levels of the neurotransmitter of interest and limited presence of other electroactive compounds. This is because electrical stimulations indiscriminately excite all proximal nerve terminals. Use of optogenetic stimulation circumvents this barrier by enabling selective excitation of a specific population of neurons. Channelrhodopsin-2-mediated serotonin efflux has been measured in fly larvae using FSCV in a technique developed by the Venton group. The light-evoked efflux is vesicular and subject to regulation by synthesis and uptake transport in a manner that is similar to mammalian serotonin release (Borue et al., 2009, 2010). Selective stimulation of serotonergic neurons in a mammalian model would permit measurements in brain regions with significant interference from other electroactive neurotransmitters, such as the hippocampus.

Finally, while voltammetric measurements of serotonin have presently only occurred in brain slices and anaesthetized animals, an exciting future direction for research will be monitoring serotonin signaling in an awake, freely moving animal. FSCV has been used to measure endogenous dopamine and norepinephrine release in freely moving animals, and this research has led to groundbreaking information coupling real-time neurotransmission to specific facets of behaviors. Many questions remain about serotonin's role in both basic and complex nervous system processes, and coupling FSCV to relevant behavioral paradigms may yield important clues about its function.

## Conclusion

**Figure 5:**
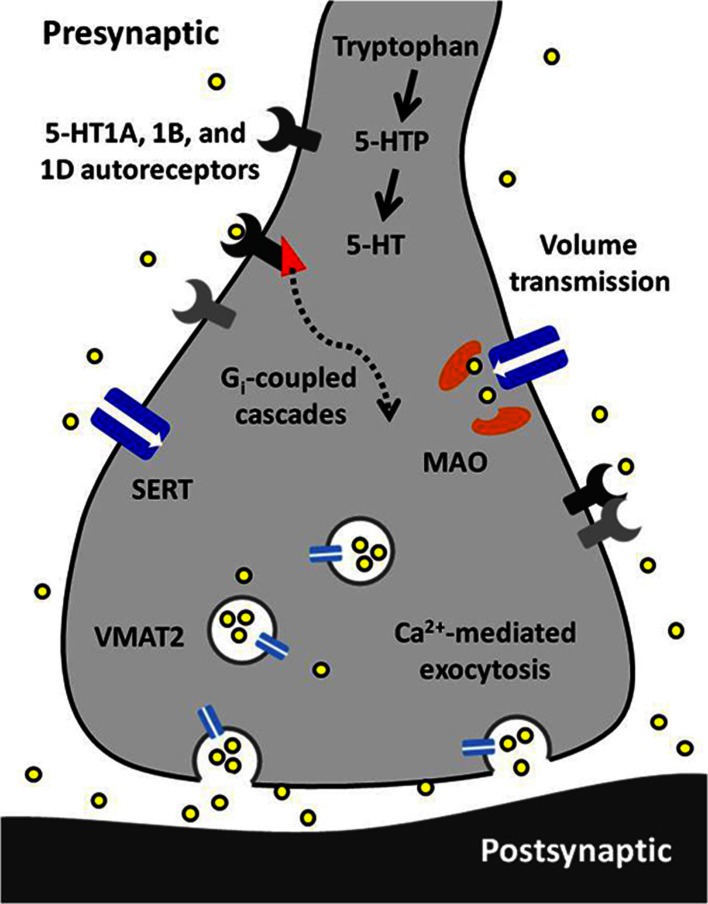
**A synopsis of the findings presented in this article**. Serotonin (5-HT) is synthesized from tryptophan in a two-step process requiring tryptophan hydroxylase and aromatic amino acid decarboxylase. Serotonin is packaged into vesicles by vesicular monoamine transporter 2 (VMAT2) and is released via calcium-dependent exocytosis. Released serotonin diffuses to extrasynaptic receptors and transporters via volume transmission. Its autoreceptors (5-HT1A, 1B, and 1D) are inhibitory and coupled to G_i_ proteins. The serotonin transporter (Thienprasert and Singer) has high affinity and selectivity for uptake of extracellular serotonin. Inside the terminal, serotonin is primarily metabolized by monoamine oxidase (MAO).

Serotonin signaling is an important component in the etiology and treatment of many neurological disorders. By combining subsecond temporal resolution with nanomolar sensitivity to concentration changes, FSCV has revealed a great deal about dynamic serotonin transmission. These findings are summarized by the illustration in Figure 5. Studies using voltammetric methods have emphasized the importance of autoreceptor-mediated inhibitory feedback mechanisms in normal signaling as well as response to SSRIs. Further, recent *in vivo* measurements suggest that intact brain circuitry supports the involvement of multiple modulatory mechanisms in the control of serotonin signaling. New developments in a variety of techniques present potential for more intricate assessment of regulation within and external to the serotonin system. Future studies using FSCV in combination with new technologies will likely elucidate many of the mysteries of the serotonin system.

### Conflict of interest statement

The authors declare that the research was conducted in the absence of any commercial or financial relationships that could be construed as a potential conflict of interest.
